# Anxiety among Lebanese adolescents: scale validation and correlates

**DOI:** 10.1186/s12887-021-02763-4

**Published:** 2021-06-22

**Authors:** Georges Merhy, Vanessa Azzi, Pascale Salameh, Sahar Obeid, Souheil Hallit

**Affiliations:** 1 Faculty of Medicine and Medical Sciences, Holy Spirit University of Kaslik (USEK), Jounieh, Lebanon; 2 INSPECT-LB: National Institute of Public Health, Clinical Epidemiology and Toxicology, Beirut, Lebanon; 3 Faculty of Pharmacy, Lebanese University, Hadat, Beirut, Lebanon; 4 University of Nicosia Medical School, Nicosia, Cyprus; 5 Faculty of Arts and Sciences, Holy Spirit University of Kaslik (USEK), Jounieh, Lebanon; 6 Research and Psychology Departments, Psychiatric Hospital of the Cross, Jal Eddib, Lebanon

**Keywords:** Anxiety, Insomnia, Child abuse, Neglect, Adolescents, Lebanon

## Abstract

**Background:**

The Lebanese population has undergone several conflicts and were the most afflicted by shelling and chaos during the civil war from 1975 to 1990, or even by displacement, bereavement, emigration, family separations, not to mention the economic crises that have hit the country since 2019 under which young adults are still succumbing. Our study aims to validate the Lebanese Anxiety Scale and assess correlates of anxiety among Lebanese adolescents.

**Methods:**

A cross-sectional study was carried between January and May 2019, using a proportionate random sampling of schools from all five Lebanese governorates, among which 1810 adolescents aged 14 to 17 years.

**Results:**

All LAS items remained in the model and formed one factor solution that explained 61.38% of the total variance (KMO = 0.873; p_Bartlett test_ < 0.001), with an excellent Cronbach’s alpha of 0.93. Higher neglect (B = 0.38), insomnia (B = 0.21) and child psychological abuse (B = 0.08) were significantly associated with more anxiety. Those results were considered adjusted overall sociodemographic variables since the latter had no statistically significant association with anxiety.

**Conclusion:**

The study confirmed the association between anxiety and some variables such as psychological child abuse, neglect, and insomnia and emphasized the correlation between anxiety and these factors. Further, the LAS appears to be a short, valid and efficient tool for assessing anxiety among Lebanese adolescents. Further studies need to be carried to evaluate whether the LAS-10 gives a similar diagnosis to psychiatrists.

## Background

According to the World Health Organization (WHO) definition, an adolescent refers to a person between 10 and 19 years old [1]. During adolescence and young adult years, adolescents are prone to mental health problems [1]. Multiple physical, emotional and social changes could predispose adolescents to mental disorders, such as the discrimination these adolescents could face during their daily life, the exclusion from a particular group, the poverty, abuse, or violence [2]. Being from a minority ethnic group or any discriminated group can as well affect the mental health of the adolescent and predispose him to mental health diseases [2]. Psychiatric conditions and disease are common in young adults, and anxiety tops the list [3]. Although anxiety is considered a normal emotion in any person, this behavior can be pathological when it is out of proportion to the stressor causing it [4]. Noting that anxiety symptoms differ from anxiety disorders which are a group of mental disorders characterized by significant feelings of anxiety and fear, as well as causing people to try to avoid situations that trigger or worsen their symptoms [5]. There are several types of anxiety disorders, including generalized anxiety disorder, panic disorder, specific phobias, agoraphobia, social anxiety disorder and separation anxiety disorder.

The DSM-5 defines general anxiety disorder as an excessive worry about particular work events or performance occurring for more than 6 months [5]. In a national face-to-face survey of 10,123 American adolescents aged 13 to 18 years, Burstein et al. (2014 & 2011) reported that around 3 and 9% of the participants had generalized anxiety disorder in 6 months duration, and social phobia in their lifetime, respectively [4, 6]. Anxiety orients us to danger; it can motivate us to face new challenges and experiences in life [7]. Many adolescents experience anxiety; almost 30% of all adolescents between 13 and 18 will have anxiety at a certain point during their adolescence [8]. Anxiety is generally expected in adolescents since teenagers have new experiences, opportunities, and challenges; hence, they seek more independence [9]. These numbers have been rising steadily; Kids and teens face much more anxiety these days [10]. Many etiologies can explain this increase in the last few years in the number of adolescents with anxiety. In fact, Dr. McCarthy, MD, believes that today’s teens are very connected to social media, unlike the last decade; therefore, their self-esteem becomes correlated to what they see on social media from posts, videos, and images [11]. Going from these concepts, we can assume that modern challenges are the primary triggers for the new generations to develop anxiety.

Different sociodemographic factors have been linked to anxiety among adolescents. Previous research reported that women and young adults, especially those under 35, are more susceptible to experience anxiety than other groups [12]. Moreover, household crowding has a robust connection with mental health issues [13]. Specifically, it has been associated with lower mental wellbeing [14].

On the other hand, higher depression scores were significantly present in adolescents who experienced physical abuse in their early years, while childhood neglect had higher anxiety scores [15]. Furthermore, there is evidence that a child sexual assault pronouncedly predisposes anxiety in early adulthood [16]. Specifically, previous literature highlighted the role of children’s physical, psychological, and sexual assaults in predisposing adults to mental health disorders such as anxiety and depression [17]. Hence, the severity of abuse or neglect was correlated with the appearance of anxiety symptoms in adulthood [17].

Additionally, the Oxford dictionary defines bullying as a way to cause harm or even intimidate others [18]. At a young age, anxiety is prevalent concurrently with bullying among involved children. A study revealed a strong association between face-to-face bullying as well as cyberbullying and anxiety [18]. Furthermore, insomnia may have bidirectional associations with anxiety disorders [19].

Moreover, previous research has demonstrated that anxiety could precipitate sleeping problems [20]. Specifically, nearly all psychiatric disorders were shown to share sleep disruption characteristics. For instance, research confirmed the robust association between chronic insomnia and anxiety [20].

When it comes to problematic internet usage, a study regarding the association between anxiety and internet addiction indicated that individuals who were highly addicted to internet usage experienced emotional, generalized anxiety, and social disorders [21].

Many scales could be used to screen for anxiety in adolescents, such as the State-Trait Anxiety Inventory for Children [22], the Revised Children’s Manifest Anxiety Scale [23], and the Fear Survey Schedule for Children-Revised [24]. Further, the developed Arab Youth Mental Health (AYMH) scale is useful as a screening tool for general mental health states and a valid screening instrument for common mental disorders among girls; however it is not a valid instrument for detecting depression and anxiety among boys in an Arab culture [25]. The LAS (Lebanese Anxiety Scale) is an approved tool to be clinically used and screen for anxiety, especially in the Lebanese population. It displays 10 items, giving more load to the anxious mood and somatic symptoms in the Lebanese population [26]. The LAS has an excellent Cronbach’s alpha when evaluating anxiety in Lebanese adults and adolescents.

Lebanon, a Middle-Eastern developing country, is facing a sudden rise in population size since 2011 due to the flow of Syrian refugees, coupled with a dysfunctional system, affecting therefore multiple sectors, particularly the economy, health, and education. Lebanon has been experiencing an unsteady political crisis, wars, armed conflicts, and terrorist attacks [27], in addition to local problems such as the shortage of clean water, electricity, and waste mishandling [27, 28]. In addition, Lebanese have been facing higher rates of unemployment especially after the displacement of a big number of Syrians after the start of the war in their country [29]. Recent studies showed that the mental health of Lebanese adolescents [30] and adults [31–33] has been affected. In addition, a previous study [34] conducted among Lebanese adolescents aged between 11 and 17 years revealed the presence of psychiatric disorders in 26.1% of those adolescents, which is higher than the numbers reported internationally [35] and in other Arab countries [36, 37]. Therefore, our study aims to validate the Lebanese Anxiety Scale and assess correlates of anxiety among Lebanese adolescents.

## Methods

### Participants

A cross-sectional study was carried between January and May 2019, using a proportionate random sampling of schools from all five Lebanese governorates, among which 1810 adolescents (90.5%) aged 14 to 17 years out of 2000 were enrolled. Additionally, a proportionate number of schools were selected from each of the five Lebanese Mohafazat (Beirut, Mount Lebanon, North, South, and Beqaa), based on The Ministry of Higher Education. A total of 18 private schools were approached; two declined while 16 agreed to partake in our study. They were distributed as follows: 4 in Beirut, 2 in South Lebanon, 6 in Mount Lebanon, 2 in North Lebanon, and 2 in Beqaa. All students between 14 and 17 years old were eligible. They had the right to accept or refuse to enroll in the study; those who agreed to participate received no financial rewards in return. Excluded were the students who refused to fill the questionnaire. The methodology used in this study is the same as in previous papers [38–41].

### Minimal sample size calculation

The Epi info software calculated a minimum sample size of 318 participants to have adequate power for the bivariate and multivariable analyses, based on 44.45% and 28.74% of anxiety disorders in adolescents with and without parental divorce, respectively [42].

### Questionnaire

The questionnaire used was anonymous, and in Lebanon’s Arabic native language, it required approximately 60 min to complete. Students filled the questionnaire at school to eliminate parents influencing interventions. Completed questionnaires were collected back and sent for data entry.

The questionnaire consisted of two parts. The first part evaluated participants’ sociodemographic characteristics, their self-reported height, their weight in order to determine the Body Mass Index (BMI), and the number of individuals and rooms in the household, excluding the bathroom and the kitchen in order to determine the household crowding index (the number of rooms divided by the number of individuals) [43]. It also collected the self-reported intensity, duration, and frequency of daily activity to calculate the Total Physical Activity Index by multiplying the three factors [44].

The second part included the following scales:

#### Lebanese anxiety scale

This 10-item scale was used to screen for the presence of anxiety [26], with higher scores indicating higher anxiety. Cronbach’s alpha in this study was 0.93.

#### Child abuse self-report scale (CASRS)

This tool includes 38 items, divided into 4 categories of child abuse: physical (8), psychological/emotional (14), sexual (5), and neglect (11). Higher scores indicated higher child abuse, evaluated as such: physical (α_Cronbach_ = 0.966), psychological (α_Cronbach_ = 0.973), sexual (α_Cronbach_ = 0.954) and neglect (α_Cronbach_ = 0.971) [45].

#### The Illinois bullying scale

This scale, validated in Lebanon [46], is used to measure bullying perpetration and victimization through a direct survey. The bullying victimization subscale was only considered in this study. Higher reported scores indicate higher bullying victimization (α_Cronbach_ = 0.975) [47].

#### Lebanese insomnia scale

This new LIS-18 scale was used to assess for the presence of insomnia [48], with higher scores indicating more insomnia. The Cronbach’s alpha was 0.742.

#### Internet addiction test

It is a self-reported tool to screen for problematic internet use among adults and adolescents. It has been previously validated in Lebanon [49, 50]. It consists of 20 items scored on a Likert-type scale ranging from 0 (Does not apply) to 5 (Always applies). The Cronbach’s alpha for this scale was 0.925.

#### Statistical analysis

The SPSS software v25 was used for data analysis. Missing data constituted < 10% of the entire database; hence, it was not replaced. Reliability was checked using Cronbach’s alpha for the total scale and its subscales. The sample was divided in half; the first was used to conduct the LAS items’ factor analysis, whereas the second was used for the confirmatory analysis. A factor analysis was initiated using the “principal component analysis” technique to confirm the legitimacy of the construct of the LAS in our sample. The Kaiser-Meyer-Olkin (KMO) value and the Bartlett’s sphericity test were checked for sampling adequacy. The factors with Eigen values > 1 were kept. The SPSS Amos software v.24 was used to conduct confirmatory factor analysis on subsample 2. Multiple indices of goodness-of-fit were described: the Relative Chi-square (χ2/df) (cut-off values:< 2-5), the Root Mean Square Error of Approximation (RMSEA) (close and acceptable fit are considered for values < 0.05 and < 0.11 respectively), the Goodness of Fit Index (GFI) and the Adjusted Goodness of Fit Index (AGFI) (acceptable values are ≥0.90) [51].

The Student t-test was used to compare two means, whereas the Pearson correlation correlates between two continuous variables. Correlation coefficients values of │0.1-0.23│, │0.24-0.36│, and > │0.37│ indicated small, moderate, and large correlations, respectively. Stepwise linear regression was finally done, taking the anxiety score as the dependent variable; independent variables entered in the linear regression were those that showed an r ≥ │0.24│ to achieve more parsimonious models [52]. R^2^ value was calculated to determine how close the data is to the fitted regression line. *P* < 0.05 was deemed statistically significant.

## Results

### Sociodemographic characteristics of the whole sample

The sociodemographic characteristics of the adolescents are summarized in Table 1. The mean age was 15.42 ± 1.14 years, with 53.3% females.

**Table 1:** Sociodemographic characteristics of the sample population (*N* = 1801)

	**Frequency (%)**
**Gender**	
Male	844 (46.7%)
Female	963 (53.3%)
**Parents status**	
Living together	1581 (88.1%)
Separated	213 (11.9%)
	**Mean ± SD**
**Age (years)**	15.42 ± 1.14
**Household crowding index**	1.01 ± 0.64

Some variables numbers do not add up to the total sample size because of missing values.

### Sociodemographic characteristics of subsample 1 (*n* = 905)

The mean age of adolescents was 15.36 ± 1.15, with 487 (53.8%) females. The mean household crowding index was 0.96 ± 0.45, whereas 165 (18.3%) of them had divorced parents.

### Sociodemographic characteristics of subsample 2 (*n* = 905)

The mean age of adolescents was 15.47 ± 1.14, with 476 (52.8%) females. The mean household crowding index was 1.05 ± 0.79, whereas 48 (5.4%) of them had divorced parents.

### Factor analysis

All LAS items remained in the model and formed one factor solution that explained 61.38% of the total variance (KMO = 0.873; p_Bartlett test_ < 0.001), with an excellent Cronbach’s alpha of 0.93. The correlation of individual items with the total score varied from 0.582 to 0.873 (*p* < 0.001 for all correlations) (Table 2).

**Table 2:** Factor analysis of the Lebanese Anxiety Scale items

Item number	Question	Factor loading	h2 communalities	Item-Total correlation
LAS 4	I have an anxious mood (Worries, anticipation of the worst, fearful anticipation, irritability)	0.876	0.768	0.873
LAS 3	I have somatic (muscular) problems (Pains and aches, twitching, stiffness, myoclonic jerks, grinding of teeth, unsteady voice, increased muscular tone)	0.851	0.724	0.854
LAS 7	I have intellectual problems (Difficulty in concentration, poor memory)	0.822	0.675	0.808
LAS 6	I have fears (of dark, of strangers, of being left alone, of animals, of traffic, of crowds)	0.816	0.666	0.810
LAS 5	I have a depressed mood (Loss of interest, lack of pleasure in hobbies, depression, early waking, diurnal swing).	0.809	0.654	0.800
LAS 2	I have tension (Feelings of tension, fatigability, startle response, moved to tears easily, trembling, feelings of restlessness, inability to relax.)	0.795	0.632	0.808
LAS 8	I feel inadequate	0.795	0.632	0.777
LAS 9	I feel that difficulties are piling up so that I cannot overcome them	0.793	0.629	0.777
LAS 1	I have insomnia (Difficulty in falling asleep, broken sleep, unsatisfying sleep and fatigue on waking, dreams, nightmares, night terrors)	0.654	0.427	0.617
LAS 10	I feel indecisive	0.575	0.331	0.582

Total variance explained = 61.38%; Cronbach’s alpha = 0.93.

### Confirmatory analysis

**Fig. 1:**
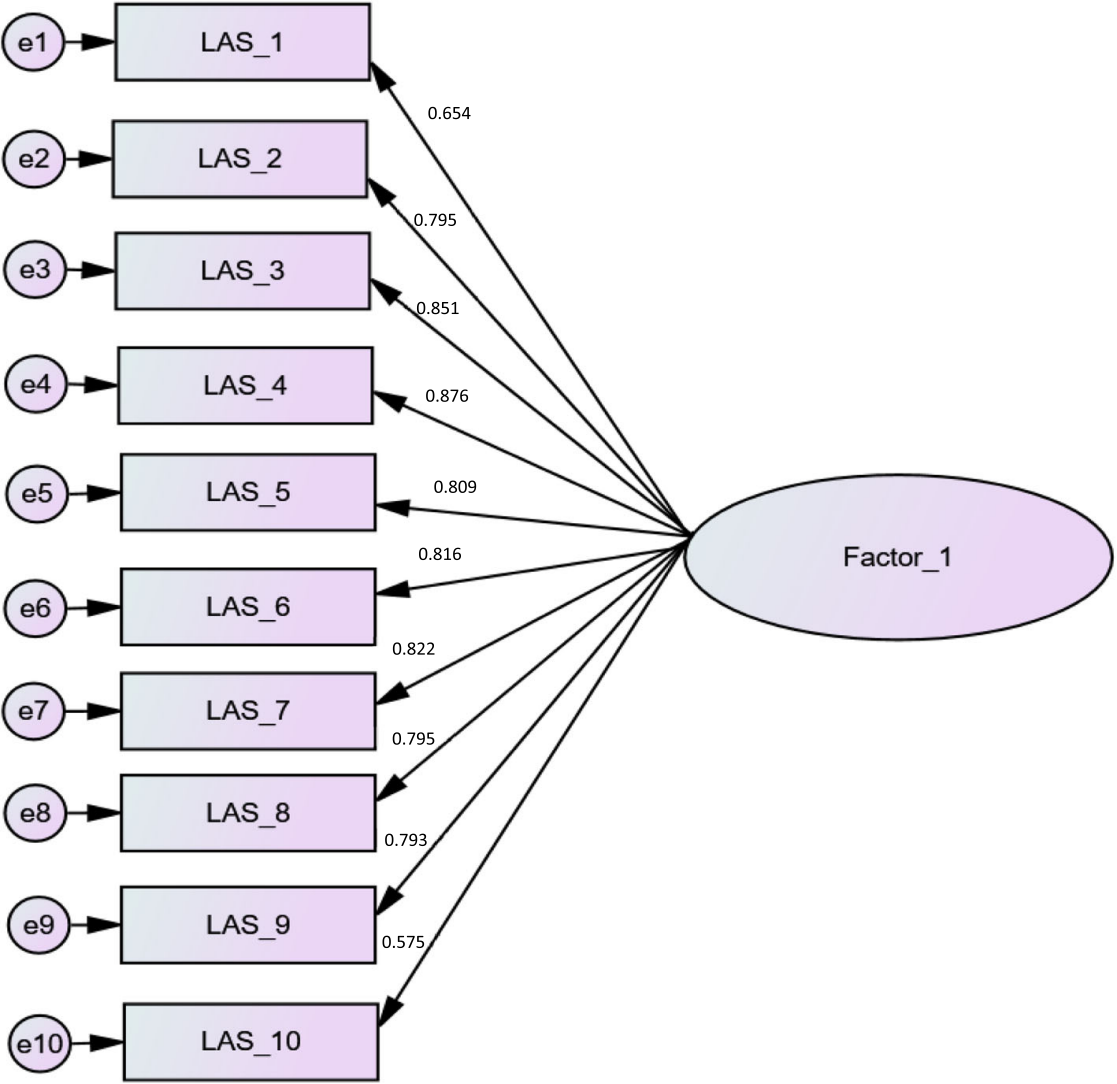
Factor loading chart of the confirmatory factor analysis of the Lebanese Anxiety Scale-10 items

A confirmatory factor analysis was run over the second subsample, using the one-factor solution obtained in the exploratory analysis. The results were as follows: the Maximum Likelihood Chi-Square = 470.82 and Degrees of Freedom = 128.64, which gave a χ^2^/df = 3.66. For non-centrality fit indices, the Steiger-Lind RMSEA was 0.082 [0.069-0.100]. Moreover, the Joreskog GFI equaled 0.93, and AGFI equaled 0.91. The factor loading chart is also summarized in Fig. 1.

### Divergent validity

Higher anxiety (higher LAS scores) was significantly correlated with higher insomnia (*r =* 0.496, *p* < 0.001), higher depression (*r =* 0.613; *p <* 0.001) and more suicidal ideation (*r =* 0.103; *p <* 0.001).

### Bivariate analysis

The results of the bivariate analysis are summarized in Tables 3 and 4. Higher neglect, child physical, sexual and psychological abuse, insomnia, and problematic internet use were significantly associated with more anxiety. None of the sociodemographic characteristics was significantly associated with anxiety.

**Table 3:** Bivariate analysis of continuous variables associated with the anxiety score

Variable	LAS score
Age	−0.03
Household crowding index	0.01
Neglect	0.55^a^
Child physical abuse	0.14^a^
Child sexual abuse	0.17^a^
Child psychological abuse	0.24^a^
Bullying victimization	0.19
Insomnia	0.68^a^
Problematic internet use	0.16^a^

^a^ *p <* 0.001; *LAS=* Lebanese Anxiety Scale

**Table 4:** Bivariate analysis of categorical variables associated with the anxiety score

Variable	LAS score
**Gender**	
Male	17.06 ± 10.66
Female	16.57 ± 8.47
*p*	0.086
Effect size	0.05
**Parents status**	
Living together	16.87 ± 10.18
Separated	17.17 ± 7.72
*p*	0.436
Effect size	0.033

*LAS* Lebanese Anxiety Scale.

### Multivariable analysis

The results of a stepwise linear regression, taking the anxiety score as the dependent variable, showed that higher neglect (B = 0.38), insomnia (B = 0.21) and child psychological abuse (B = 0.08) were significantly associated with more anxiety (Table 5, Model 2). Those results were considered adjusted overall sociodemographic variables since the latter had no statistically significant association with anxiety.

**Table 5:** Multivariable analysis: Stepwise linear regression taking the anxiety score as the dependent variable

**Model 1: Parental divorce as an independent variable**				
**Variable**	**UB**	**SB**	***p***	**95% CI**
Parental status (separated vs living together^a^)	−0.85	− 0.03	0.185	−2.10-0.41
**Model 2: Parental divorce and other scales as independent variables**				
**Variable**	**UB**	**SB**	***p***	**95% CI**
Neglect	0.38	0.46	**< 0.001**	0.35-0.42
Insomnia	0.21	0.27	**< 0.001**	0.18-0.24
Child psychological abuse	0.08	0.09	**< 0.001**	0.04-0.11

^a^Reference group; Numbers in bold indicate significant *p*-values; *R*^2^ = 42.6%; variables entered in model 1: parental divorce; Variables entered in model 2: parental divorce, neglect, insomnia, child psychological abuse. *UB* Unstandardized Beta; *SB* Standardized Beta, *CI* Confidence Interval

## Discussion

This cross-sectional study involving 1810 adolescents from 16 schools across all the five Lebanese governorates showed the robust association between anxiety in adolescents and the following variables: neglect, insomnia, and child psychological abuse. Furthermore, it validated the Lebanese Anxiety Scale among this age group, with an excellent Cronbach’s alpha of 0.93 in this study.

The results of the exploratory factor analysis of the LAS in this study showed a convergence of the scale’s items to a one-factor solution, showing a more solid construction than with adults, where the ten items converged into two factors. The items’ internal reliability was higher in adolescents (0.93) compared to adults (0.857). The EFA results were even confirmed by the results of the confirmatory factor analysis. The LAS-10 appears to be a promising tool that may be clinically used and screen for anxiety in adults and adolescents [26].

Child neglect was significantly associated with higher anxiety in our study, while previous corroborating findings showed that neglect was as well correlated with anxiety [53]. In fact, it was proven that neglect significantly affects the anatomic development of the brain structures [54]. A study on the correlation between self-reported neglect, amygdala volume, and anxiety indicated that neglect was significantly associated with boys’ right amygdala volume [54]. Additional analyses also revealed that the right amygdala volume is a mediator of this association [54]. Therefore, the results suggest that there was some effect on the limbic structures, which may, at a certain point, explain the physiological association between neglect and anxiety.

When it comes to psychological abuse, our results’ analysis showed that higher psychological abuse was significantly associated with more anxiety. In fact, child psychological abuse has been associated with many organic and psychiatric disorders such as depression, anxiety disorders, post-traumatic stress disorder, eating disorders, irritable bowel syndrome, and also somatic symptoms [55]. Although child psychological abuse was correlated with anxiety in adolescence and adulthood, the pathophysiology behind it is not still well understood [55].

The association between insomnia and anxiety has also been well demonstrated in this study. As such, anxiety is frequently connected to sleeping problems in general, and more frequently, to insomnia. A few mental and physiological variables contribute to the onset and propagation of sleep deprivation, such as anxious-ruminative identity characteristics, upsetting occasions, age-related sleep homeostasis, debilitating mechanisms, and numerous other components [56]. Researchers also affirm that sleep deprivation can cause anxiety as well [57].

### Clinical implications

Anxiety can be diagnosed clinically by the physician or the psychiatrist based on the Diagnostic and Statistical Manual of Mental Disorders, 5th Edition (DSM-5). Recognizing associated factors is the beginning point for identifying and anticipating anxiety among vulnerable adolescents and treating symptoms. Besides, wellbeing care experts are advised to spread mindfulness to diminish the predominance of these components. Screening, anxiety monitoring, and treatment intercession among youths are also prescribed.

### Limitations

Despite having a sample of 1810 Lebanese adolescents, the questionnaire was answered subjectively by the students, leading to information bias since some of the students could exaggerate or underestimate some facts while answering personally to the questionnaire. Thus, anxiety was not confirmed by a physician (a professional psychiatrist). Some psychometric characteristics of the LAS scale are still missing (convergent validity and comparison to a physician’s diagnosis). In addition to that, some students may view things in a subjective, inaccurate way because of cultural backgrounds. For instance, some students who have already experienced psychological or physical abuse will not report in the questionnaire, considering such behavior normal towards them. Hence, such behavior could lead to information bias. On another hand, few students may forget some details of sexual or any form of neglect or abuse. In this case, we might have to face a recall bias while collecting data in the questionnaire. A selection bias is also possible due to the refusal rate and public schools that were not enrolled. A residual confounding bias is conceivable since other factors are associated with anxiety that was not considered in this paper. In addition to that, the results cannot be generalized to the whole Lebanese adolescent population.

## Conclusion

The study confirmed the association between anxiety and some variables such as psychological child abuse, neglect, and insomnia and emphasized the correlation between anxiety and these factors. Further, the LAS appears to be a short, valid and efficient tool for assessing anxiety among Lebanese adolescents. Further studies need to be carried to evaluate whether the LAS-10 gives a similar diagnosis to psychiatrists.

## Data Availability

The authors have the right to share the database following a reasonable request to the corresponding author.

## Abbreviations

LAS: Lebanese Anxiety Scale
BMI: Body Mass Index
CASRS: Child abuse self-report scale
KMO: Kaiser-Meyer-Olkin
DSM-5: Diagnostic and Statistical Manual of Mental Disorders, 5th Edition
